# Impact of Increasing Capacity for Generating and Using Research on Maternal and Perinatal Health Practices in South East Asia (SEA-ORCHID Project)

**DOI:** 10.1371/journal.pone.0023994

**Published:** 2011-09-07

**Authors:** The SEA-ORCHID Study Group

**Affiliations:** University of Sydney, Australia

## Abstract

**Background:**

Maternal and neonatal mortality and morbidity remain unacceptably high in many low and middle income countries. SEA-ORCHID was a five year international collaborative project in South East Asia which aimed to determine whether health care and health outcomes for mothers and babies could be improved by developing capacity for research generation, synthesis and use.

**Methods:**

Nine hospitals in Indonesia, Malaysia, the Philippines and Thailand participated in SEA-ORCHID. These hospitals were supported by researchers from three Australian centres. Health care practices and outcomes were assessed for 1000 women at each hospital both before and after the intervention. The capacity development intervention was tailored to the needs and context of each hospital and delivered over an 18 month period. Main outcomes included adherence to forms of care likely to be beneficial and avoidance of forms of care likely to be ineffective or harmful.

**Results:**

We observed substantial variation in clinical practice change between sites. The capacity development intervention had a positive impact on some care practices across all countries, including increased family support during labour and decreased perineal shaving before birth, but in some areas there was no significant change in practice and a few beneficial practices were followed less often.

**Conclusion:**

The results of SEA-ORCHID demonstrate that investing in developing capacity for research use, synthesis and generation can lead to improvements in maternal and neonatal health practice and highlight the difficulty of implementing evidence-based practice change.

## Introduction

### Context

Improving maternal mortality is the Millennium Development Goal towards which the least progress has been made globally [1]. Despite a 20% reduction in maternal deaths in Asia since 1990, it is estimated that over 240,000 women still die in childbirth each year in the region compared to fewer than 1000 in developed regions [2]. The main causes of maternal mortality directly related to pregnancy and childbirth are bleeding, infection, hypertension (including eclampsia) obstructed labour and unsafe abortions [3]. Serious acute and chronic maternal morbidity has been estimated to occur in one in four women [4]. Poor maternal health and pregnancy care leads to high rates of perinatal death and also to low birth weight, perinatal asphyxia and infection [5]. Many of these outcomes could be avoided through provision of simple, effective, evidence-based care before, during and after birth [1].

To improve the quality of health care, low and middle income countries need to develop local capacity to use, synthesise and generate research relevant to their health needs [6]. This capacity is vital to ensure that relevant research is undertaken, identified, disseminated and implemented, so ensuring that optimal care is provided.

‘Capacity’ refers to appropriate resources, skills, commitment and structure [7]. Capacity development therefore requires more than just provision of technical assistance, but rather a multifaceted approach that includes:

- access to research resources
- training and skills development in doing, finding, understanding and using research
- development of sustainable networks within and between organisations and countries to support a culture of research, research synthesis and research use
- structures that enable each of the above.

We aimed to build on existing links with the Cochrane Collaboration and International Clinical Epidemiology Network to implement and evaluate a targeted capacity development intervention in South East Asia.

We hypothesised that a targeted intervention to build capacity for generating, synthesising and implementing relevant evidence would lead to improved adherence to appropriate clinical practices leading to better health outcomes for women and babies in South East Asia.

This paper describes the changes in clinical practices and outcomes in the participating hospitals following the intervention phase of the SEA-ORCHID (South East Asia – Optimising Reproductive and Child Health in Developing Countries) Project.

## Methods

### Objective

The SEA-ORCHID project aimed to assess whether the health of mothers and babies in Thailand, Indonesia, the Philippines and Malaysia could be improved by increasing capacity for the synthesis of relevant research, implementation of effective interventions, and identification of gaps in knowledge needing further research [8].

To achieve this aim, the objectives of the SEA-ORCHID project were:

1. to build capacity in research synthesis
2. to increase capacity and skills for evidence-based practice
3. to demonstrate effective implementation strategies for evidence-based practice change
4. to improve access to quality healthcare information
5. to increase locally derived and relevant research activity
6. to increase evidence-based policy making
7. to influence the broader socio-economic and health policy environment [9].

### Design

SEA-ORCHID was a before-after study, using action research to design, tailor and implement a capacity development intervention which included skills development (training and support), network building (training fellowships and project meetings), mentoring (between-country exchanges) and resourcing (subscription to evidence based materials, travel to project meetings and relevant conferences, IT infrastructure and support).

The SEA-ORCHID project protocol, methods and design, and data on clinical practices at baseline have been published previously [8], [9], [10].

### Ethics Statement

The project was approved by the appropriate local ethics committee for each hospital (THAILAND: Khoen Kaen University Ethics Committee for Human Research for Srinagarind Khon Kaen University Hospital; Ethical Review Committee for Research in Human Subjects, Ministry of Public Health for Khon Kaen Regional Hospital and Kalasin General Hospital; THE PHILIPPINES: Research Implementation & Development Office, University of the Philippines Manila for Philippine General Hospital, Manila; Hospital Epidemiology & Research Unit, Dr Jose Fabella Memorial Hospital, Department of Public Health for Dr. Jose Fabella Memorial Hospital, Manila; MALAYSIA: Ethics Committee, Ipoh Hospital for Ipoh Hospital, Perak; School of Medical Sciences, Universiti Sains Malayisa for Universiti Sains Malaysia, Kota Bharu; INDONESIA: Ethical Committee of Research in Medical Health, Ministry of Education, Faculty of Medicine, Gadjah Mada University for Dr. Sardjito Hospital, Yogyakarta and Sleman District Hospital)and by the ethics committee of the administering institution in Australia (University of Sydney). Individual patient informed consent was not required for quantitative data collection as all data was collected by audit of medical records.

### Setting

**Figure 1:**
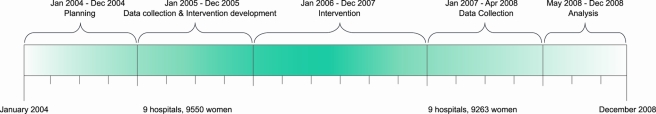
SEA-ORCHID Project Timeline.

The SEA-ORCHID project was conducted between 2004 and 2009 (see Figure 1) in nine hospitals across Indonesia, Malaysia, The Philippines and Thailand with support from three universities in Australia. Seven of the hospitals were tertiary referral institutions with regional referrals of women with a high risk pregnancy. Two hospitals were provincial or district institutions. In all hospitals, vaginal births were conducted by doctors, obstetric specialists and/or midwives (including nurses with midwifery qualifications) with caesarean section facilities available.

### Population

Data were collected on at least 1000 women at each site pre and post-intervention. This sample size was based on the maximum possible recruitment at the smaller participating hospitals. The five smaller hospitals collected data on consecutive women until at least 1000 women were included. The four larger hospitals sampled women using appropriate ratios to ensure there was at least a three-month data collection period and avoid biases associated with short data collection periods. At pre-intervention, 9550 women (9665 infants including 111 twins and two sets of triplets) presenting to one of the labour wards at each site were enrolled in the study and their medical records audited by trained researchers to determine compliance with the practices of interest. At post-intervention, the medical records of 9263 women (9351 infants including 84 twins and two sets of triplets) were audited using the same methods. The time taken to enrol 1000 women per site varied according to the number of births per month. Due to this, in the post-intervention phase, enrolment at the two Indonesian sites began in August, 2007, several months before the other sites.

### Intervention

The intervention was implemented for 24 months (January 2006–December 2007). Tailored intervention strategies aiming to increase capacity in generating, synthesising and using research were implemented at each hospital based on local need and evidence for their effectiveness in leading to change in the maternal and child healthcare practices. The intervention was iteratively refined using action research methods [9].

The intervention included:

- Training based around a fellowships program (see below), teaching tours of SE Asian nodes and project meetings. Training focused on activities for three target groups:
  - Generators of research evidence and evidence-based materials: training emphasising critical appraisal, systematic reviewing and guideline development.
  - Users of the evidence: training for clinicians (i.e. doctors, nurses, midwives, etc) in maternal and neonatal units of participating hospitals in accessing,interpreting and implementing evidence.
  - Educators about evidence: training for clinical trainers and opinion leaders covering facilitation of practice change, adult education methods, principles of evidence-based practice.
- Systematic reviewing
  We identified relevant interventions for the management of pregnancy and childbirth in SE Asia for which systematic review evidence was lacking. Reviews were prepared and published in The Cochrane Library, and the results actively disseminated.
- Guideline development
  We aimed to facilitate locally relevant evidence-based guideline development and implementation.
- Access to research
  Provision of computers, internet access, subscriptions to The Cochrane Library and other evidence-based information sources and IT support.
- Academic exchange
  More than 20 fellowships in Australia were provided to researchers and clinicians from the maternal and neonatal units of participating institutions in SE Asia, and the Australian educators travelled to the nodes to conduct workshops and partner the SE Asian trainers in the development of materials [11].
- Promotion
  Information about evidence- based practice, the SEA-ORCHID project and the results of the research conducted was published in the academic literature and presented at local, national and international meetings, conferences and Cochrane Colloquia.
- Input into the undergraduate curriculum
  We facilitated teaching of evidence-based practice skills in medical, nursing and allied health schools by sharing knowledge, skills and materials from Australia.

Further details are available on the SEA-ORCHID website (www.seaorchid.org).

### Main outcome measures

The primary outcomes were changes in maternal and perinatal care practices during the time of the SEA-ORCHID project intervention. These included adherence to forms of care likely to be beneficial and avoidance of forms of care likely to be ineffective or harmful drawn from The Cochrane Library and the World Health Organization Reproductive Health Library No. 7 (see Table 1).

**Table 1:** Recommended practices in maternal and perinatal health care.

Recommended practice	Outcome intended to reduce
**Forms of care likely to be beneficial**	
Antibiotics for preterm prelabour rupture of membranes (pPROM) [19]	Chorioamnionitis; neonatal sepsis
Magnesium sulphate for eclampsia and pre-eclampsia [20], [21], [22]	Maternal death; eclampsia
Corticosteroids prior to preterm birth [23]	Neonatal death; complications of preterm birth
External cephalic version for breech presentation at term [24]	Caesarean section rate; birth trauma
Continuous support during labour [12]	Caesarean section rate
Vacuum extraction (versus forceps) for operative delivery [25]	Perineal injury; postpartum haemorrhage
Intraoperative antibiotics during caesarean section [26]	Maternal infection
Active management of third stage of labour including: [27]• Appropriate administration of a prophylactic oxytocic at or after birth of the baby• Early cord clamping and cutting• Controlled cord traction to deliver the placenta	Postpartum haemorrhage; maternal death
Perineal suture material and technique [28], [29]	Maternal infection
Immunisation for Hepatitis B [30]	Hepatitis B infection
**Forms of care likely to be harmful**	
Routine shaving* [31]	Maternal infection
Routine enemas* [32]	Maternal infection
Routine episiotomy [13]	Perineal injury; maternal infection

*No clear evidence from Cochrane reviews to support or refute use, but identified by South East Asian teams as practices of importance to research and evaluate.

The secondary outcomes were measures of maternal and perinatal morbidity and mortality, including:

- Maternal outcomes
  1. maternal death, eclampsia, perineal trauma, caesarean section rate, postpartum haemorrhage and postpartum pyrexia
- Perinatal outcomes
  1. perinatal death, preterm birth (gestational age <37 weeks), low birth weight (<2500 grams), small for gestational age (SGA) and perinatal asphyxia (Apgar score <7 at 5 minutes).

### Data collection

Data were collected during the following two intervals: pre-intervention (*baseline*) during January–December, 2005 and post-intervention (*endpoint*) during January–June, 2008. Manually completed data extraction forms were sent to the country project office for entry into a secure web-based data management system.

### Statistical analyses

We used descriptive analyses to describe maternal and infant characteristics for the pre- and post-intervention cohorts across countries and hospitals. Frequencies were used to describe categorical data variables such as nulliparity, preterm birth and caesarean section, and means and standard deviations to describe the continuous data variables of maternal age, gestational age at birth and birth weight. Differences in beneficial and harmful practices, and health outcomes between pre- and post-intervention were presented using risk differences and 95% confidence intervals (CI).

We categorised maternal age to be <20, 20–34 and ≥35 years and gestational age to be preterm (<37 weeks) and term (≥37 weeks). Potential confounding factors, including maternal age, gestational age, parity and caesarean rates were evaluated for their association with each health outcome in hospitals using chi-square test at the 5% level of significance. All health outcome results were adjusted for gestational age and parity. For each health outcome, risk differences and confidence intervals (RD, 95% CI) were adjusted for any other significant associated factors in the individual country data set using Mantel-Haenzel method. Analyses were done at the Thai site using Stata (version 10.0).

## Results

Mean maternal age and nulliparous rate were similar between pre- and post-intervention periods, as were preterm birth and low birth weight. Caesarean section rates were higher in the post-intervention period in the participating hospitals in all four countries (Table 2).

**Table 2:** Characteristics of women and infants sampled in pre and post-intervention periods.

Characteristics	Period	Indonesia	Malaysia	Philippines	Thailand
**No. of mothers**	Pre	**2086**	**2379**	**2085**	**3000**
	Post	**2014**	**2249**	**2000**	**3000**
**No. of infants**	Pre	**2113**	**2410**	**2098**	**3044**
	Post	**2043**	**2271**	**2007**	**3030**
**Maternal age (years)** *	Pre	**30 (5.5)**	**30 (6.2)**	**27 (6.4)**	**27 (6.1)**
	Post	**30 (5.8)**	**30 (6.1)**	**28 (6.5)**	**27 (6.1)**
**Nulliparous (%)**	Pre	**46**	**31**	**48**	**58**
	Post	**50**	**32**	**52**	**59**
**Caesarean section (%)**	Pre	**30**	**19**	**23**	**35**
	Post	**37**	**27**	**29**	**43**
**Preterm birth** (<37 wks) **(%)**	Pre	**10**	**10**	**7**	**11**
	Post	**10**	**11**	**7**	**7**
**Low birth weight** (<2500 gm) **(%)**	Pre	**18**	**11**	**20**	**11**
	Post	**18**	**13**	**22**	**10**

*Mean and (standard deviation).

Some care practices were largely in line with the evidence at baseline. Following the capacity development intervention, alignment between clinical practices and best evidence improved in many cases. However, in some cases there was no significant change in practice and there were decreases in a few potentially beneficial practices.

### Forms of care likely to be beneficial

#### Practices during the antenatal period (Table 3)

**Table 3:** Percentage of women receiving forms of care likely to be beneficial during the antenatal period.

		INDONESIA			MALAYSIA			THE PHILIPPINES			THAILAND			
Hospitals	Period	Overall	Tertiary	District	Overall	Tertiary 1	Tertiary 2	Overall	Tertiary 1	Tertiary 2	Overall	Regional	University	Provincial
**MgSO4 for eclamptic fit**	Pre	**100**	100	100	**100**	100	–	**83**	100	80	**100**	100	–	–
	Post	**100**	100	100	**100**	–	100	**33**	100	0	**100**	100	–	–
**Diff (95%CI)**		**0**			**0**			**−50 (−111.1, 11.1)**			**0**			
**Antibiotic use for pPROM**	Pre	**91**	88	100	**62**	83	44	**92**	99	67	**73**	91	55	63
	Post	**96**	100	67	**75**	70	79	**79**	83	75	**79**	100	65	64
**Diff (95%CI)**		**5.2 (−13.3, 23.8)**			**12.3 (−3.7, 28.3)**			**−12.7 (−21.8, −3.7)**			**6.1 (−7.2, 19.5)**			
**MgSO4 for pre-eclampsia**	Pre	**100**	100	100	**24**	44	13	**86**	91	84	**64**	57	82	67
	Post	**90**	92	77	**65**	76	37	**60**	51	66	**69**	53	95	82
**Diff (95%CI)**		**−10.5 (−15.9, −5.1)**			**40.6 (24, 57.2)**			**−25.2 (−36.8, −13.6)**			**5.1 (−8.8, 19)**			
**Corticosteroids given to women who gave birth at <34 wks** (1)	Pre	**12**	11	17	**68**	67	69	**15**	0	21	**82**	88	88	65
	Post	**37**	38	25	**91**	87	95	**31**	10	37	**86**	100	75	89
**Diff (95%CI)**		**24.8 (11, 38.7)**			**23.4 (6.6, 40.2)**			**16.5 (−0.3, 33.3)**			**3.7 (−9.6, 16.9)**			
**ECV offered** (for breech at > = 37 wks)(2)	Pre	**0**	0	0	**9**	15	4	**2**	0	3	**3**	2	0	8
	Post	**53**	93	0	**19**	21	16	**5**	6	5	**37**	33	0	75
**Diff (95%CI)**		**53 (45, 61)**			**9.9 (1, 18.8)**			**3.3 (−1.7, 8.4)**			**33.6 (25.5, 41.8)**			
**ECV performed** (for breech at > = 37 wks)	Pre	**0**	0	0	**4**	9	0	**2**	0	3	**3**	2	0	6
	Post	**19**	34	0	**7**	11	3	**2**	0	3	**1**	0	0	2
**Diff (95%CI)**		**19.5 (13.1, 25.8)**			**2.9 (−3, 8.8)**			**−0.3 (−4, 3.5)**			**−1.9 (−4.7, 0.9)**			

(1) number of women receiving corticosteroids at GA 24–33 wks/number of women giving birth during GA 24–33 wks (Note: denominator for Post intervention excluded congenital malformations, DFIU, BBA, septic criminal abortion, denominator for Pre and Post intervention excluded stillbirth).

(2) May not be recorded accurately.

There was moderate to high use of antibiotics for women with preterm prelabour rupture of the membranes (pPROM) both at baseline (range 62–92%) and post-intervention (range 75–96%) in line with recommended practice. There was however, a significant decrease in the use of antibiotics for pPROM in the participating hospitals in the Philippines from 92 to 79% (RD −13, 95% CI −22 to −4).

The use of magnesium sulphate for eclampsia was 100% both pre- and post-intervention in the participating hospitals in Indonesia, Malaysia and Thailand. At baseline the use of magnesium sulphate for women with pre-eclampsia varied (range 24–100%). There was a significant increase in the use of magnesium sulphate for pre-eclampsia in the Malaysian sites (24 to 65%, RD 41, 95% CI 24–57) but a significant decrease in the sites in Indonesia (100 to 90%, RD −10, 95% CI −16 to −5) and the Philippines (86 to 60%, RD −25, 95% CI −37 to −14).

Antenatal corticosteroids for women who gave birth before 34 weeks gestation were infrequently used in sites in Indonesia and the Philippines (12% and 15% respectively) at baseline. Post-intervention there were significant increases in the proportion of women given antenatal corticosteroids for this indication in the Indonesian (12 to 37%, RD 25, 95% CI 11–39) and Malaysian (68 to 91%, RD 23, 95% CI 7–40) hospitals.

External cephalic version (ECV) for women with a breech presentation at term was rarely used in any of the hospitals at baseline (range 0–4%). Post-intervention ECV was offered to women significantly more frequently in Malaysia (9 to 19%, RD 10, 95% CI 1–19) and Thailand (3 to 37%, RD 34, 95% CI 26–42). However, actually performing an ECV only increased significantly in the Indonesian hospitals (0 to 20%, RD 20, 95% CI 13–26).

#### Practices during the intrapartum period (Table 4)

**Table 4:** Percentage of women receiving forms of care likely to be beneficial during the intrapartum and postpartum period.

		INDONESIA			MALAYSIA			THE PHILIPINES			THAILAND			
Hospitals	Period	Overall	Tertiary	District	Overall	Tertiary 1	Tertiary 2	Overall	Tertiary 1	Tertiary 2	Overall	Regional	University	Provincial
**Appropriate prophylactic oxytocic given during 3rd stage** (1)	Pre	**26**	26	26	**5.4**	10	1	**27.4**	<1	64	**58**	4	76	98
	Post	**9**	14	3	**7.7**	8	8	**47.6**	63	27	**91**	99	78	95
**Diff (95%CI)**		**−17 (−19.7, −14.3)**			**2.3 (0.6, 3.9)**			**20.2 (16.7, 23.5)**			**32.8 (30.2, 35.3)**			
**Controlled cord traction to deliver the placenta**	Pre	**100**	100	100	**100**	100	99	**98**	100	96	**56**	31	43	96
	Post	**100**	100	100	**99**	100	99	**93**	93	92	**90**	97	71	99
**Diff (95%CI)**		**−0.3 (−0.7, 0)**			**−0.4 (−1, 0.1)**			**−5.2 (−6.7, −3.7)**			**33.9 (31.3, 36.6)**			
**Family support during labour** (2)	Pre	**41**	38	45	**61**	86	32	**10**	<1	19	**53**	83	31	44
	Post	**80**	100	65	**65**	67	64	**49**	63	36	**73**	95	63	62
**Diff (95%CI)**		**38.8 (35.9, 41.7)**			**4.9 (2.1, 7.7)**			**38.9 (36.3, 41.5)**			**20.7 (18.3, 23.1)**			
**Appropriate antibiotic use for caesarean** (3)	Pre	**0**	0	0	**<1**	<1	0	**5**	0	6	**49**	72	82	<1
	Post	**0**	0	0	**<1**	0	<1	**38**	72	23	**40**	4	80	27
**Diff (95%CI)**		**0**			**−0.1 (−0.6, 0.5)**			**33.2 (28.7, 37.6)**			**−9.2 (−13.2, −5.2)**			
**Early cord clamping and cutting**	Pre	**100**	100	100	**100**	100	100	**96**	100	92	**100**	99	100	100
	Post	**100**	100	100	**100**	100	100	**59**	48	74	**71**	92	100	19
**Diff (95%CI)**		**0 (−0.3, 0.3)**			**0.1 (−0.1, 0.2)**			**−37.3 (−40.1, −34.6)**			**−28.7 (−30.9, −26.6)**			
**Vacuum extraction** (4)	Pre	**100**	100	100	**83**	80	85	**0**	0	0	**77**	84	60	93
	Post	**100**	100	100	**66**	73	52	**0**	0	0	**94**	94	84	100
**Diff (95%CI)**		**0**			**−16.7 (−32.8, −0.7)**			**0**			**17.3 (10.1, 24.4)**			
**Perineal suture material** (5)	Pre	**0**	0	0	**0**	0	0	**3**	0	5	**2**	0	6	0
	Post	**<1**	0	<1	**28**	1	61	**3**	1	4	**5**	0	15	<1
**Diff (95%CI)**		**0.1 (−0.1, 0.3)**			**28.4 (26, 30.8)**			**0 (−1.3, 1.4)**			**2.8 (1.6, 4)**			
**Perineal suture technique** (6)	Pre	**60**	61	60	**72**	68	77	**44**	3	88	**100**	100	100	100
	Post	**30**	23	36	**54**	72	33	**57**	34	84	**99**	100	99	98
**Diff (95%CI)**		**−30.4 (−34.2, −26.6)**			**−17.6 (−21.1, −14.1)**			**13.1 (9, 17.2)**			**−0.8 (−1.3, −0.3)**			
**Immunisation for Hep B** (7)	Pre	**2**	<1	3	**100**	100	100	**2**	<1	4	**99**	99	100	99
	Post	**38**	40	36	**98**	99	97	**64**	66	62	**99**	99	99	100
**Diff (95%CI)**		**36.2 (34, 38.4)**			**−1.7 (−2.3, −1.1)**			**62.6 (60.2, 64.9)**			**0.2 (−0.3, 0.6)**			

(1) Defined as administration of oxytocin or syntocin at anterior shoulder or after birth (denominator is total vaginal birth),

(2) Defined as the proportion of husbands, mothers, sisters, other family members or friends giving either “some/little” or “all/most” support. For post-intervention the time period is first stage of labour,

(3) Defined as given a single dose of ampicillin or cephalosporin after cord clamped (denominator is total caesarean),

(4) Vacuum/vacuum + forceps,

(5) Rate of use polyglycolic acid suture material (where perineum sutured),

(6) Rate of continuous skin closure (where perineum sutured),

(7) Excluded stillbirths.

Post-intervention, family support during labour was practiced significantly more frequently in the hospitals in all four countries. The highest increases, with similar risk differences of approximately 39% (95% CI 36–42), were observed in the hospitals in Indonesia and the Philippines. The pre-intervention practice rate was highest in the Indonesian hospitals (49%).

The use of vacuum extraction for assisted vaginal delivery significantly increased post-intervention in the hospitals in Thailand (77 to 94% RD 17, 95% CI 10–24) but significantly decreased in Malaysia (83 to 66%, RD −17 95% CI-33 to −1).

Appropriate use of prophylactic antibiotics for caesarean section was close to zero in both the pre- and post-intervention periods in the Indonesian and Malaysian hospitals. A significant increase was observed post-intervention in the hospitals in the Philippines (5 to 38%, RD 33, 95% CI 29–38), but there was a significant decrease in Thailand (49 to 40%, RD −9, 95% CI −13 to −5).

There were significant increases in the post-intervention use of an appropriate prophylactic oxytocics during the third stage of labour in the hospitals in Malaysia (5 to 8%, RD 2.2, 95% CI 1–4), the Philippines (27 to 48%, RD 20, 95% CI 17–24) and Thailand (58 to 91%, RD 33, 95% CI 30–35), but a significant decrease in Indonesia (26 to 9%, RD −17, 95% CI −20 to −14).

Early cord clamping and cutting was almost universally practiced in pre- and post-intervention periods in the participating hospitals in Indonesia and Malaysia. Significantly lower levels of practice were observed post-intervention in the Philippines (96 to 59%, RD −37 95% CI −40 to −35) and Thailand (100 to 71%, RD −9 95% CI −31 to −27), in keeping with newer recommendations.

Controlled cord traction to deliver the placenta was almost universally practiced in pre and post intervention periods in the Indonesian and Malaysian hospitals. Post-intervention practice significantly decreased in the Philippines (98 to 93%, RD −5 95% CI −7 to −4) and significantly increased in the Thai hospitals (56 to 90%, RD 34 95% CI 31–37).

Appropriate perineal suture technique significantly increased post-intervention in the hospitals in the Philippines (44 to 57%, RD 13 95% CI 9–17) but significantly decreased in Indonesia (60 to 30%, RD −30 95% CI −34 to −27) and Malaysia (72 to 54%, RD −18 95% CI −21 to −14). The use of appropriate suture materials (polyglycolic) significantly increased post-intervention in Malaysia (0 to 28%, RD 28 95% CI 26–31) and Thailand (2 to 5%, RD 3 95% CI 2–4).

#### Practices during postpartum period

There were significant increases in immunisation for hepatitis B in the hospitals in Indonesia (2 to 38%, RD 36, 95% CI 34–38) and the Philippines (2 to 64%, RD 63, 95% CI 60–65). Pre and post-intervention immunisation rates for hepatitis B were high in the hospitals in Thailand (100%) and Malaysia (99%).

### Forms of care likely to be harmful (Table 5)

**Table 5:** Percentage of women receiving forms of care likely to be harmful.

		INDONESIA			MALAYSIA			THE PHILIPINES			THAILAND			
Hospitals	Period	Overall	Tertiary	District	Overall	Tertiary 1	Tertiary 2	Overall	Tertiary 1	Tertiary 2	Overall	Regional	University	Provincial
**Episiotomy for vaginal births**	Pre	**54**	49	60	**46**	61	31	**64**	64	64	**91**	95	93	86
	Post	**50**	41	60	**41**	52	30	**62**	55	72	**84**	82	96	74
**Diff (95%CI)**		**−4 (−7.7, −0.2)**			**−5.5 (−8.8, −2.2)**			**−1.7 (−5.2, 1.9)**			**−7.7 (−9.8, −5.5)**			
**Pubic hair shaving**	Pre	**28**	26	29	**33**	24	42	**26**	12	40	**74**	43	79	98
	Post	**0**	1	0	**24**	0	51	**22**	9	35	**42**	17	83	27
**Diff (95%CI)**		**−27.3 (−29.2, −25.3)**			**−8.7 (−11.2, −6.1)**			**−3.9 (−6.6, −1.3)**			**−31.4 (−33.8, −29)**			
**Enema use**	Pre	**17**	17	18	**22**	2	44	**1**	<1	2	**30**	1	29	61
	Post	**0**	0	0	**23**	0	49	**0**	0	0	**6**	0	17	1
**Diff (95%CI)**		**−17.2 (−18.9, −15.6)**			**1.1 (−1.3, 3.5)**			**−1.2 (−1.7, −0.6)**			**−24.1 (−25.9, −22.2)**			

Perineal hair shaving before birth was significantly less likely post-intervention across all four countries. The largest decrease was observed in the Thai hospitals (74 to 42%, RD −32 95% CI −34 to −29).

Enema use significantly decreased post-intervention in the hospitals in Indonesia, the Philippines and Thailand, which showed the biggest decrease (30 to 6%, RD −24 95%CI −26, −22).

Routine episiotomy for vaginal births significantly decreased post-intervention in the hospitals in Indonesia (54 to 50%, RD −4 95% CI −8 to −0.2), Malaysia (46 to 41%, RD −5 95% CI −9 to −2) and Thailand (91 to 84%, RD −8 95% CI −10 to −6). Despite this, the practice was still frequent in the post-intervention period across hospitals in all countries, especially in Thailand.

### Maternal and perinatal outcomes (Table 6)

**Table 6:** Maternal and perinatal outcomes.

		INDONESIA		MALAYSIA		THE PHILIPPINES		THAILAND	
Maternal outcomes	Period	% (n/N)	adj RD (95% CI)	% (n/N)	adj RD (95% CI)	% (n/N)	adj RD (95% CI)	% (n/N)	adj RD (95% CI)
**Maternal death**	Pre	0 (0/2086)	n/a	0 (0/2379)	n/a	0 (0/2084)	n/a	0 (0/3000)	n/a
	Post	0.1 (3/2014)		0 (1/2249)		0 (0/1999)		0 (0/3000)	
**Eclampsia** (1)	Pre	0.3 (7/2086)	**0.97 (0.02, 1.93)**	0 (1/2378)	−0.18 (−0.94, 0.57)	0.3 (6/2071)	0.10 (−0.47, 0.68)	0.1 (3/3000)	0.04 (−0.34, 0.43)
	Post	1.3 (27/2014)		0 (1/2249)		0.2 (3/1981)		0.1 (2/2999)	
**Intact perineum** ∧ (2)	Pre	13 (188/1451)	0.34 (−2.56, 3.25)	19.9 (381/1919)	1.26 (−1.7, 4.22)	20.8 (254/1224)	**13.69 (3.5, 23.89)**	3 (58/1953)	1.23 (−0.56, 3.02)
	Post	12.5 (157/1259)		18.4 (302/1645)		33.1 (433/1309)		4.8 (82/1714)	
**Caesarean section** (2)	Pre	29.6 (618/2086)	**7.19 (1.57, 12.81)**	19.1 (454/2376)	**7.56 (2.92, 12.2)**	22.7 (474/2085)	**5.53 (0.58, 10.48)**	34.8 (1045/3000)	**7.88 (3.12, 12.63)**
	Post	37.5 (755/2014)		26.9 (604/2249)		28.7 (573/2000)		42.7 (1280/3000)	
**PPH** (>500 mls) ∧ (2)	Pre	1 (15/1468)	0.47 (−1.3, 2.24)	1.3 (24/1920)	1.3 (−0.55, 3.15)	48.4 (695/1436)	−**18.4 (−25.5, −11.4)**	1.2 (24/1954)	0.45 (−1.15, 2.04)
	Post	1.6 (20/1259)		2.5 (41/1643)		30.5 (412/1351)		1.9 (33/1720)	
**Severe PPH** (>1000 mls) ∧ (3)	Pre	0.4 (6/1468)	−0.04 (−0.95, 0.87)	0.4 (7/1920)	0.12 (−0.58, 0.81)	0.8 (12/1436)	0.47 (−1.04, 1.97)	0.2 (4/1954)	0.18 (−0.56, 0.93)
	Post	0.6 (8/1259)		0.4 (6/1643)		1.4 (19/1351)		0.4 (7/1720)	
**Postpartum pyrexia - vaginal birth** (2)	Pre	0 (0/1468)	n/a	0.4 (7/1913)	0.15 (−0.55, 0.85)	0.1 (2/1564)	0.32 (−0.5, 1.13)	3.4 (66/1955)	−2.7 (−5.67, 0.28)
	Post	0 (0/1259)		0.7 (8/1219)		0.7 (10/1415)		1.3 (23/1720)	
**Postpartum pyrexia - caesarean section** (2)	Pre	0 (0/617)	1.12 (−1.35, 3.6)	1.3 (6/454)	0.82 (−1.53, 3.17)	1.8 (8/448)	−1.0 (−3.58, 1.58)	9.7 (101/1044)	**−6.8 (−10.7, −2.9)**
	Post	0.4 (3/755)		2 (12/597)		0.7 (4/568)		3 (38/1279)	

Perinatal Outcomes	Period	% (n/N)	adj RD (95% CI)	% (n/N)	adj RD (95% CI)	% (n/N)	adj RD (95% CI)	% (n/N)	adj RD (95% CI)
**Low birthweight** (<2500 g) (4)	Pre	18 (380/2113)	0.08 (−3.45, 3.61)	11.1 (267/2403)	0.42 (−2.32, 3.17)	19.9 (414/2085)	1.39 (−2.9, 5.67)	11.4 (347/3043)	0.27 (−1.85, 2.39)
	Post	17.5 (358/2042)		12.5 (285/2271)		21.9 (439/2003)		9.8 (298/3030)	
**Preterm birth** (<37 wks) (5)	Pre	10.2 (215/2113)	−0.71 (−4.25, 2.84)	9.9 (238/2396)	0.77 (−2.66, 4.2)	7.3 (151/2063)	−0.23 (−3.34, 2.88)	11 (335/3043)	**−4.47 (−7.26, −1.68)**
	Post	9.7 (198/2039)		11.2 (254/2266)		7.5 (150/2001)		6.5 (197/3030)	
**SGA** * (6)	Pre	31.6 (666/2110)	−3.89 (−9.32, 1.55)	20.1 (480/2391)	−1.22 (−5.65, 3.21)	33.1 (683/2061)	3.8 (−2.02, 9.61)	18 (546/3041)	−1.91 (−5.54, 1.71)
	Post	27.9 (569/2038)		18.9 (428/2266)		37 (739/1999)		15.9 (483/3030)	
**Birth asphyxia** (Apgar <7 at 5 mins) (4)	Pre	8.1 (171/2113)	0.59 (−1.92, 3.1)	1.4 (33/2402)	0.13 (−0.67, 0.93)	1.3 (28/2077)	−0.2 (−0.87, 0.47)	1.8 (55/3044)	−0.25 (−1.13, 0.62)
	Post	8.8 (179/2043)		1.6 (34/2146)		1.1 (22/1988)		1.7 (51/3030)	
**Perinatal death** ** (4)	Pre	3.5 (74/2113)	1.44 (−0.3, 3.19)	1.2 (28/2410)	0.15 (−0.56, 0.86)	0.4 (8/2097)	0.53 (−0.62, 1.68)	1.6 (49/3044)	−0.13 (−0.84, 0.58)
	Post	4.1 (84/2043)		1.4 (32/2271)		1.2 (24/2005)		1.6 (49/3030)	

* calculation of SGA by using Australian birth charts,

** perinatal death = stillbirth+death before discharge,

∧ vaginal birth,

(1) Adjusted for parity (P), caesarean section (C/S), gestational age (GA).

(2) Adjusted for P, maternal age (MA), GA.

(3) Adjusted for P, GA.

(4) Adjusted for P, C/S, MA, GA.

(5) Adjusted for P, C/S.

(6) Adjusted for P, C/S, MA.

There were no significant differences in the rates of maternal death, severe postpartum haemorrhage or postpartum pyrexia after vaginal birth between pre- or post-intervention. Caesarean rates significantly increased across the hospitals in all four countries (see Table 2). There were small statistically significant variations in postpartum haemorrhage after vaginal birth, intact perineum and pyrexia after caesarean section at some sites, but no consistent pattern.

There were no significant differences in low birth weight, small for gestational age, birth asphyxia and perinatal death detected between pre- and post-intervention periods.

**Figure 2:**
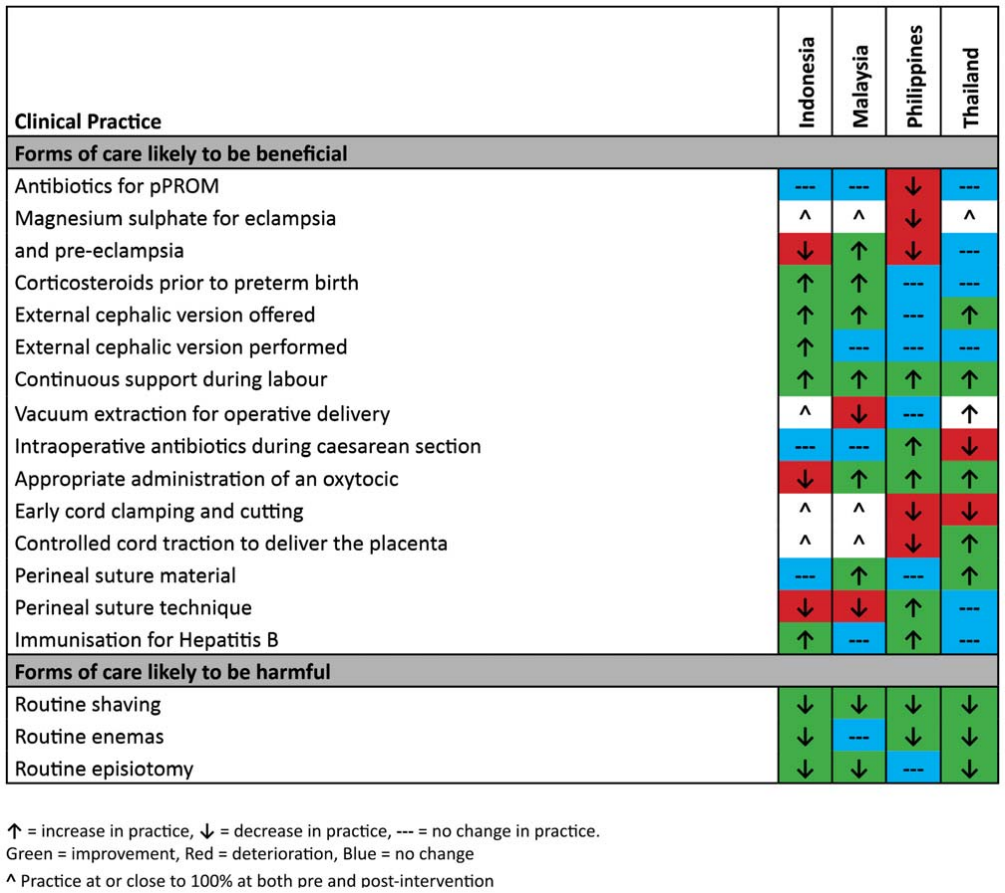
Summary of changes in clinical practice.

A summary of all the changes in clinical practice is provided in Figure 2.

## Discussion

SEA-ORCHID was a large, complex, multi-site project which involved several countries and a substantial sample of women. The outcomes were assessed at the level of the patient, with clinical practices and health outcomes evaluated through file audit by trained staff. The participating sites included provincial, regional and university hospitals. The project was truly collaborative with the centre of gravity of the research in the South East Asian countries, led by teams of local clinicians and researchers supported from Australia. The project built on the existing networks developed through the Cochrane Collaboration and International Clinical Epidemiology Network. The intervention aimed to develop sustainable capacity for individuals rather than simply provide technical support and was tailored to meet local needs and be appropriate to the local context. The intervention was multidisciplinary; targeting doctors, nurses, midwives and non-clinical staff including librarians and biostatisticians. Importantly, SEA-ORCHID was resourced to deliver a complex, multi-faceted capacity development project over five years.

The focus of the project was examining whether an intervention to develop capacity for research generation, synthesis and use could result in improvements in healthcare practice. We viewed capacity development in terms of knowledge and skills (training, mentoring, fellowships), resources, leadership and commitment. We believe our intervention had a substantial impact on improving knowledge and skills related to using, synthesising and generating research and resulted in a supportive network of clinical leaders with expertise and commitment to evidence-based practice; this in turn led to some changes in clinical practice. The project was not designed to overcome more structural/systemic barriers to change such as limited availability of drugs or suture materials, shortages of skilled staff, and this is evidenced in our limited impact on practices that were dependent on these factors.

Our study demonstrates that investment in capacity development for research can lead to changes in clinical practice in a broad range of clinical environments. The nature of the SEA-ORCHID intervention which focused on capacity development and relied on investment in knowledge and skills rather than infrastructure or equipment, makes it likely that similar approaches could be effective in other clinical areas and others settings, both low and high resource. The design of the project, which encouraged tailoring of the intervention to address local needs and barriers, also increases its generalisability.

SEA-ORCHID had some limitations. Randomised controlled trials (RCTs) are the most reliable design for assessing the impact of an intervention as they minimise selection bias and confounding. A cluster-RCT design could have been used for this project, with hospitals as clusters. However, the number of clusters included would have been severely limited by the project resources and timelines, leading to substantial risk of baseline imbalance, particularly as there is wide variation in clinical practice and other baseline variables between hospitals. In light of this, a before-and-after design was chosen, a design which was optimal for the project purpose, and the timeframe and funding available.

We were powered to demonstrate an effect on healthcare practices and not maternal or neonatal health outcomes as the resources and time available did not make it feasible to recruit the sample size required to demonstrate an impact on health outcomes. However the healthcare practices examined have been demonstrated to impact on health outcomes. Increased rates of family support have been shown to decrease duration of labour, increase rates of normal vaginal birth and decrease need for analgesia [12]. Similarly our reported decreased rates of episiotomy have the potential to improve health outcomes by reducing perineal trauma and preventing complications [13].

The impact of SEA-ORCHID on other clinical practices varied widely between participating countries and hospitals, reflecting the ways in which these sites differed in terms of culture, infrastructure, and resources. It is likely that the impact of SEA-ORCHID on many of these practices may also be seen over a longer timeframe, reflecting the time required for improvements in knowledge to translate into changes in practice. The barriers to and enablers of each of these changes is being explored through a qualitative evaluation.

Despite being well resourced and comprising a large and experienced research team with excellent local and international connections, the observed improvements to clinical practice were limited. This reflects both the difficulties involved in implementing evidence-based practice changes, and the emphasis of this project on developing research capacity rather than clinical skills. The results of the forthcoming qualitative analysis of barriers and enablers of practice change at the participating hospitals may provide some insights into the reasons for variation between hospitals as well as highlight opportunities for further improvements in practice. In a similar study of four hospitals in China that investigated the impact of national and local initiatives to promote evidence-based obstetric care, the influence of other factors, such as hospital directors and legislation, were deemed to be especially important in changing provider behaviour [14]. A recent study in three hospitals in Jordan highlighted that the need to identify effective methods to support evidence-based maternal healthcare practice remains an imperative [15] and the results of SEA-ORCHID also suggest that there is still much to be learnt about effective knowledge translation strategies in this context.

Over the course of the SEA-ORCHID project there was an increase in the overall caesarean section rate. We believe this change is reflective of a global increase in caesarean section rate [16]. A change from secondary to tertiary status at some of the participating hospitals during the project and subsequent increase in their high risk patient profile may also have influenced this.

In addition to the clinical practice and health outcomes reported in this paper we have also assessed a range of intermediate outcomes at the participating sites. These outcomes include randomised controlled trials, systematic review and clinical practice guideline development, and awareness and understanding of evidence-based practice (for examples see [17], [18]). The impact of SEA-ORCHID on these outcomes will be reported in detail elsewhere, but the findings are consistent with the hypothesis that increasing capacity in these areas can lead to improved clinical practice.

The capacity built through SEA-ORCHID will enable practice at participating hospitals to keep pace with research discoveries and facilitate a research-informed culture. We observed this during the project when the evidence about the best practice in management of third stage of labour changed. Pleasingly, several centres modified their practice in line with this new evidence, demonstrating their capacity to understand and apply evidence appropriately. It is also likely that the impact of the SEA-ORCHID project will extend beyond maternal and perinatal health care and into practice in other clinical areas.

While we no longer have resources to invest in the training or to support travel and fellowships, we are hopeful that the network developed through the project will be maintained and will lead to ongoing relationships, mentoring, and research collaborations. We are exploring opportunities for shared projects and are encouraging involvement in the Cochrane Collaboration as a vehicle to sustain the relationships developed.

If we are to reach the Millennium Development Goals related to health we need to substantially invest in capacity development for generating, synthesising and using research in LMICs, in order to improve healthcare practice. This paper has demonstrated that such an investment can have an impact on improving healthcare practice and contribute to this goal.

## References

[1] United Nations (2009). The Millennium Development Goals Report.

[2] Hill K, Thomas K, AbouZahr C, Walker N, Say L (2007). Estimates of maternal mortality worldwide between 1990 and 2005: an assessment of available data.. Lancet.

[3] Sauvarin J (2006). Maternal and Neonatal Health in East and South-East Asia.

[4] Razmjou H, Davis AM, Jaglal SB, Holtby R, Richards RR (2011). Disability and satisfaction after Rotator Cuff decompression or repair: a sex and gender analysis.. BMC Musculoskeletal Disorders.

[5] Save the Children (2006). State of the world's newborns report.

[6] Global Forum for Health Research (2008). Fostering innovation for global health: Global Forum Update on Research for Health.

[7] NSW Health (2001). A Framework for Building Capacity to Improve Health.

[8] Henderson-Smart DJ, Lumbiganon P, Festin MR, Ho JJ, Mohammad H (2007). Optimising reproductive and child health outcomes by building evidence-based research and practice in South East Asia (SEA-ORCHID): study protocol.. BMC Med Res Methodol.

[9] McDonald S, Turner T, Chamberlain C, Lumbiganon P, Thinkhamrop J (2010). Building capacity for evidence generation, synthesis and implementation to improve the care of mothers and babies in South East Asia: methods and design of the SEA-ORCHID Project using a logical framework approach.. BMC Med Res Methodol.

[10] Laopaiboon M, Lumbiganon P, McDonald SJ, Henderson-Smart DJ, The SEA-ORCHID Study Group (2008). Use of evidence-based practices in pregnancy and childbirth: South East Asia Optimising Reproductive and Child Health in Developing Countries project.. PLoS ONE.

[11] Short J, McDonald S, Turner T, Martis R (2010). Improving capacity for evidence-based practice in South East Asia: evaluating the role of research fellowships in the SEA-ORCHID Project.. BMC Med Educ.

[12] Hoddnet E, Gates S, Hofmeyr G, Sakala C (2007). Continuous support for women during childbirth.. Cochrane Database Syst Rev.

[13] Carroli G, Belizan J (2009). Episiotomy for vaginal birth.. Cochrane Database Syst Rev.

[14] Qian X, Smith H, Liang H, Liang J, Garner P (2006). Evidence-informed obstetric practice during normal birth in China: trends and influences in four hospitals.. BMC Health Serv Res.

[15] Shaban IA, Hatamleh R, Khresheh R, Homer C (2011). Childbirth practices in Jordanian public hospitals: consistency with evidence-based maternity care?. Int J Evid Based Healthc.

[16] Stanton CK, Holtz SA (2006). Levels and trends in cesarean birth in the developing world.. Stud Fam Plann.

[17] Suwannachat B, Laopaiboon M, Tonmat S, Siriwachirachai T, Teerapong S (2011). Rapid versus stepwise application of negative pressure in vacuum extraction-assisted vaginal delivery: a multicentre randomised controlled non-inferiority trial.. BJOG.

[18] Suwannachat B, Lumbiganon P, Laopaiboon M (2008). Rapid versus stepwise negative pressure application for vacuum extraction assisted vaginal delivery.. Cochrane Database Syst Rev.

[19] Kenyon S, Boulvain M, Neilson J (2003). Antibiotics for preterm rupture of membranes.. Cochrane Database Syst Rev.

[20] Duley L, Henderson-Smart D (2003). Magnesium sulphate versus diazepam for eclampsia.. Cochrane Database Syst Rev.

[21] Duley L, Henderson-Smart D (2003). Magnesium sulphate versus phenytoin for eclampsia.. Cochrane Database Syst Rev.

[22] Duley L, Gulmezoglu AM, Henderson-Smart DJ (2003). Magnesium sulphate and other anticonvulsants for women with pre-eclampsia.. Cochrane Database Syst Rev.

[23] Roberts D, Dalziel S (2006). Antenatal corticosteroids for accelerating fetal lung maturation for women at risk of preterm birth.. Cochrane Database Syst Rev.

[24] Hofmeyr GJ (2004). Interventions to help external cephalic version for breech presentation at term.. Cochrane Database Syst Rev.

[25] Johanson R, Menon V (1999). Vacuum extraction versus forceps for assisted vaginal delivery.. Cochrane Database of Systematic Reviews.

[26] Smaill F, Hofmeyr G (2002). Antibiotic prophylaxis for cesarean section.. Cochrane Database Syst Rev.

[27] Prendiville WJ, Elbourne D, McDonald S (2000). Active versus expectant management in the third stage of labour.. Cochrane Database Syst Rev.

[28] Kettle C, Hills RK, Ismail KM (2007). Continuous versus interrupted sutures for repair of episiotomy or second degree tears.. Cochrane Database Syst Rev.

[29] Kettle C, Johanson RB (2000). Absorbable synthetic versus catgut suture material for perineal repair.. Cochrane Database Syst Rev.

[30] Lee C, Gong Y, Brok J, Boxall EH, Gluud C (2006). Hepatitis B immunisation for newborn infants of hepatitis B surface antigen-positive mothers.. Cochrane Database Syst Rev.

[31] Basevi V, Lavendar T (2000). Routine perineal shaving on admission to labour.. Cochrane Database Syst Rev.

[32] Cuervo L, Rodriguez M, Delgado M (1999). Enemas during labour.. Cochrane Database Syst Rev.

